# Survival Benefit of Temozolomide Plus Irinotecan as Second-Line Therapy in Small Cell Lung Cancer: A Retrospective Single-Center Study

**DOI:** 10.3390/jcm14207287

**Published:** 2025-10-15

**Authors:** Omer Acar, Ahmet Burak Agaoglu, Mustafa Sahbazlar, Ferhat Ekinci, Atike Pınar Erdogan

**Affiliations:** 1 Department of Medical Oncology, Mardin Training and Research Hospital, Vali Ozan Street, Artuklu, Mardin 47100, Turkey; 2 Department of Medical Oncology, Manisa Celal Bayar University, Manisa 45030, Turkey

**Keywords:** small cell lung cancer, temozolomide, irinotecan, second-line therapy, overall survival, progression-free survival

## Abstract

**Background:** Small cell lung cancer (SCLC) is an aggressive type of cancer known for its rapid progression and poor prognosis. While several chemotherapeutic agents, including topotecan, are approved for use in the second-line treatment setting, their clinical benefits have been modest and often limited by toxicity. As a result, there is a significant need for more effective treatment strategies. Given the high rate of brain metastases in patients with SCLC and temozolomide’s (TMZ) ability to penetrate the central nervous system, combining TMZ with irinotecan (IRI) presents a potentially effective therapeutic approach. This study aimed to evaluate the clinical outcomes of the TMZ and IRI combination compared to other second-line treatment regimens in a real-world patient population. **Methods:** We conducted a retrospective review of the medical records of 37 patients with relapsed SCLC who underwent second-line therapy at a tertiary oncology center from January 2018 to December 2023. Among these patients, 24 were treated with a combination of TMZ+IRI, while 13 received alternative regimens, which included topotecan, irinotecan, paclitaxel, docetaxel, vinorelbine, or gemcitabine. We collected baseline demographic and clinical data and assessed survival outcomes. Overall survival (OS) and progression-free survival (PFS) were estimated using the Kaplan–Meier method, and prognostic factors were analyzed using Cox regression models. **Results:** A total of 37 patients were included (mean age 59.7 years, 86.5% male). Baseline characteristics were similar between groups, except for body mass index, which was higher in the TMZ+IRI group (27.9 vs. 24.6, *p* = 0.033). Median OS was significantly longer in patients treated with TMZ+IRI compared to controls (25 vs. 8 months, *p* = 0.002). One-year OS rates were 58.2% and 25.4%, respectively. In multivariate analysis, brain metastases (HR 0.37, 95% CI 0.14–0.95, *p* = 0.039) and receipt of non-TMZ+IRI regimens (HR 2.82, 95% CI 1.03–7.72, *p* = 0.044) were independent predictors of poor OS. Median PFS did not differ significantly between groups (8 vs. 7 months, *p* = 0.733), and no independent predictors of PFS were identified. **Conclusions:** The combination of temozolomide and irinotecan was associated with a significant overall survival benefit compared with other second-line regimens in relapsed SCLC, despite similar progression-free survival. These findings suggest that TMZ+IRI may provide a clinically meaningful option for appropriately selected patients, particularly those with preserved performance status. Prospective randomized studies are warranted to confirm these results and better define the role of this regimen in treatment sequencing.

## 1. Introduction

Small cell lung cancer (SCLC) makes up about 13–15% of all lung cancer cases and is known for its aggressive progression, quick tumor doubling, and early widespread metastasis [1]. Most patients present with extensive-stage disease at the time of diagnosis. While first-line treatment with platinum-based chemotherapy and etoposide (with or without immune checkpoint inhibitors) often results in high initial response rates, relapse is nearly unavoidable. Consequently, the prognosis for SCLC remains poor, with a median overall survival (OS) of just 8–13 months for extensive-stage disease and a 5-year survival rate below 7% [2,3]. After disease progression after first-line therapy, second-line treatment options are limited and generally lead to poor outcomes. For example, topotecan’s modest clinical efficacy, hematologic toxicity, and restricted use in patients with poor performance status limit its use. Real-world data report a median OS of only ~5.6 months in SCLC patients treated with topotecan outside of clinical trials [4]. Other agents such as lurbinectedin, irinotecan, and temozolomide, paclitaxel, and docetaxel are being used as possible second-line treatments, but none have yet been established as standard of care in global guidelines [5,6,7,8,9].

Temozolomide, an oral alkylating agent approved for glioblastoma, has shown activity in SCLC because it can cross the blood–brain barrier and cause DNA alkylation-mediated cell death [10]. Irinotecan, a topoisomerase I inhibitor, has demonstrated notable activity as a salvage option in later-line treatment for patients with small cell lung cancer who have progressed after first-line therapy [11]. Temozolomide causes DNA damage through alkylation and readily crosses the blood–brain barrier, while irinotecan inhibits DNA replication by targeting topoisomerase I. While effective as monotherapy in relapsed SCLC, the combination may offer a mechanistically complementary approach, as irinotecan can potentiate the DNA damage caused by temozolomide. This potential synergy, along with the favorable tolerability and activity reported in other solid tumors such as Ewing sarcoma, supports the rationale for testing this regimen in relapsed SCLC, particularly in patients with brain metastases [12,13,14]. Brain metastases frequently develop in patients with SCLC and are a significant cause of both morbidity and mortality. Because temozolomide can effectively penetrate the central nervous system, combining it with irinotecan may be especially beneficial for this high-risk group.

A thorough literature review found no prospective, randomized, or large-scale phase II/III studies that investigate the combination of temozolomide and irinotecan as a treatment option for relapsed SCLC, including patients with brain metastases. While several studies have examined temozolomide or irinotecan as individual treatments, there is a significant lack of robust evidence systematically assessing the combination of temozolomide and irinotecan in this context. Therefore, our study is unique in its exploration of the tolerability, central nervous system penetration, and therapeutic efficacy of this combination in relapsed SCLC. In this retrospective cohort study, we aimed to evaluate the clinical effectiveness of temozolomide plus irinotecan as a second-line treatment for patients with SCLC. Specifically, we compared survival outcomes between this combination and other commonly used regimens. Additionally, we examined the prognostic value of key clinical and demographic variables, including performance status, BMI, and metastatic pattern, on survival outcomes in this population. Our results contribute to the expanding body of real-world evidence aimed at defining optimal sequencing strategies in the second-line management of SCLC.

## 2. Methods

### 2.1. Study Design and Patient Selection

This retrospective cohort study was conducted at the Medical Oncology Department of Manisa Celal Bayar University Hospital. The medical records of patients diagnosed with SCLC who were treated at the outpatient oncology clinic between January 2018 and December 2023 were retrospectively reviewed.

**Figure 1:**
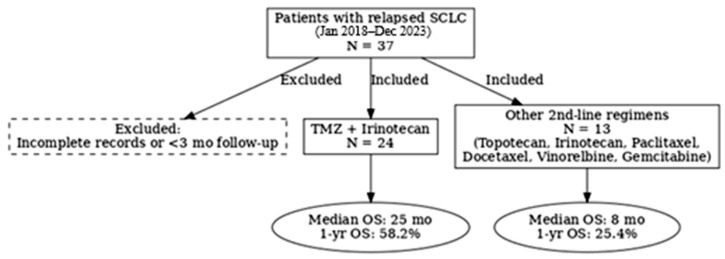
Flow diagram of patient selection and treatment allocation. Between January 2018 and December 2023, a total of 37 patients with relapsed SCLC who received second-line therapy were identified. Of these, 24 received temozolomide plus irinotecan (TMZ+IRI) and 13 received alternative regimens, including topotecan, irinotecan, paclitaxel, docetaxel, vinorelbine, or gemcitabine. Patients with incomplete records or follow-up shorter than 3 months were excluded. OS, overall survival.

Eligible patients were those who: (1) had histologically confirmed SCLC, (2) were ≥18 years of age, (3) had received second-line systemic therapy following disease progression after platinum-based chemotherapy, and (4) had available follow-up and clinical data. Patients treated with TMZ+IRI were compared with those who received other second-line regimens. Patients with incomplete records or a follow-up duration of less than 3 months were excluded (Figure 1).

### 2.2. Treatment Regimens

Patients in the TMZ+IRI group received temozolomide orally at a dose of 150–200 mg/m^2^ on days 1–5 of each 28-day cycle. Irinotecan was administered intravenously at a dose of 150 mg/m^2^ once every two weeks (days 1 and 15 of each cycle). Treatment doses and schedules were determined according to institutional standards and physician discretion based on patient tolerance and clinical status. Patients in the control group received alternative second-line regimens, including topotecan, irinotecan, paclitaxel, docetaxel, vinorelbine, gemcitabine, or temozolomide, at the discretion of the treating oncologist.

### 2.3. Data Collection

Data were extracted from the institutional electronic health records. Demographic and clinical variables included age at diagnosis, sex, smoking status (never, former, current), body mass index (BMI), Eastern Cooperative Oncology Group (ECOG) performance status, comorbidities (e.g., diabetes mellitus, hypertension, coronary artery disease, chronic kidney disease), site of metastases (brain, liver, lung, bone, others), radiotherapy exposure, and the number of treatment lines received.

### 2.4. Outcomes

The primary endpoint was OS, defined as the time from the start of second-line treatment to death from any cause. The secondary endpoint was PFS, defined as the time from initiation of second-line treatment to the first documented radiological or clinical progression or death, whichever occurred first.

### 2.5. Statistical Analysis

Statistical analyses were performed using IBM SPSS Statistics version 25.0 (IBM Corp., Armonk, NY, USA). Categorical variables were summarized as frequencies and percentages and compared using the chi-square test or Fisher’s exact test. Continuous variables were presented as means ± standard deviation (SD) or medians with interquartile ranges (IQR), depending on distribution. Group comparisons were performed using Student’s t-test or the Mann–Whitney U test, as appropriate. Survival analyses were conducted using the Kaplan–Meier method and compared between groups using the log-rank test. Cox proportional hazards regression models were used for univariate and multivariate analysis to identify predictors of OS and PFS. Results were reported as hazard ratios (HR) with 95% confidence intervals (CI). A *p*-value of <0.05 was considered statistically significant.

### 2.6. Ethical Approval

This study was approved by the Ethics Committee of Manisa Celal Bayar University (approval number: 2026, dated 11 October 2023). All procedures were performed in accordance with the principles of the Declaration of Helsinki. As this was a retrospective study, the requirement for informed consent was waived.

## 3. Results

### 3.1. Patient Characteristics

A total of 37 patients who received second-line therapy for SCLC were included, with 24 in the TMZ+IRI group and 13 in the control group (treated with topotecan, irinotecan, paclitaxel, docetaxel, vinorelbine, or gemcitabine monotherapy). Baseline demographic and clinical features were comparable between groups (Table 1). The mean age at diagnosis was 59.7 years, and the majority of patients were male (86.5%) and current or former smokers. Median BMI was significantly higher in the TMZ+IRI group compared to the control group (27.9 vs. 24.6, *p* = 0.033). Other baseline variables, including ECOG performance status, comorbidities, and metastatic distribution, were similar between groups.

### 3.2. Overall Survival

**Figure 2:**
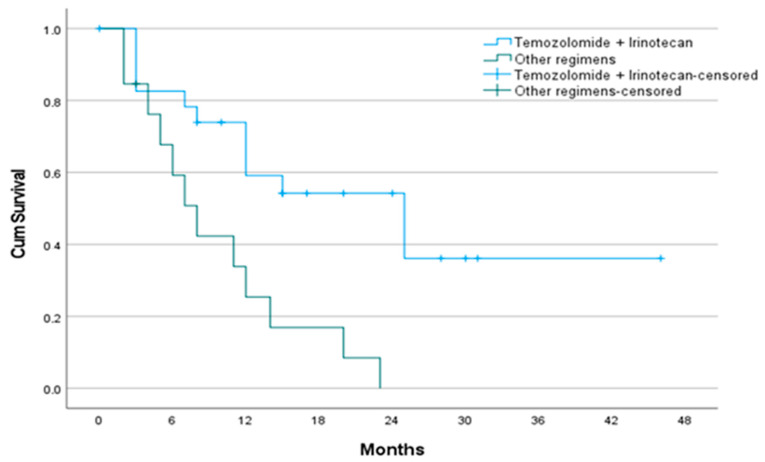
Kaplan–Meier curve for overall survival according to second-line treatment. Overall survival was significantly longer in the temozolomide plus irinotecan group compared with other regimens (25 vs. 8 months, *p* = 0.002). Other regimens; topotecan, irinotecan, paclitaxel, docetaxel, vinorelbine, or gemcitabine.

The median follow-up duration was 12 months (IQR, 4.5–20). At the time of analysis, 64.9% of patients had died. Median OS was significantly longer in the TMZ+IRI group compared to the controls (25 vs. 8 months, *p* = 0.002) (Table 2, Figure 2). One-year OS was 58.2% in the TMZ+IRI group versus 25.4% in the control group. In univariate Cox regression analysis, lower BMI, ECOG ≥ 2, and receipt of non-TMZ+IRI regimens were associated with worse OS. In multivariate analysis, the presence of brain metastases (HR 0.37, 95% CI 0.14–0.95, *p* = 0.039) and use of alternative second-line regimens (HR 2.82, 95% CI 1.03–7.72, *p* = 0.044) remained independent predictors of poor OS (Table 3).

### 3.3. Progression-Free Survival

**Figure 3:**
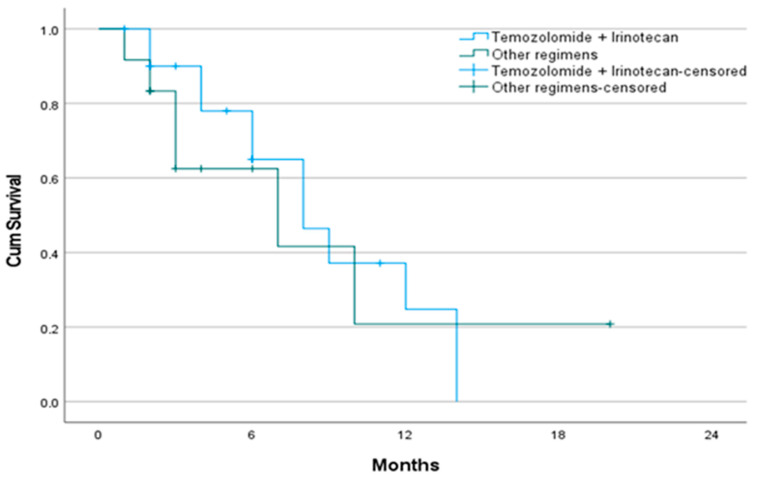
Kaplan–Meier curve for progression-free survival according to second-line treatment. No significant difference in progression-free survival was observed between the temozolomide + irinotecan and control groups (8 vs. 7 months, *p* = 0.733). **Other regimens**: topotecan, irinotecan, paclitaxel, docetaxel, vinorelbine, or gemcitabine.

Median PFS was 8 months in the TMZ+IRI group and 7 months in the control group (*p* = 0.733) (Table 4, Figure 3). Six-month PFS rates were similar between groups (62.5% vs. 65.0%). No statistically significant predictors of PFS were identified in multivariate analysis (Table 5).

## 4. Discussion

The management of relapsed SCLC remains a major therapeutic challenge, as most patients experience rapid disease progression after first-line platinum–etoposide therapy and derive only limited benefit from available second-line regimens [1,2,3]. In this retrospective cohort study, the combination of temozolomide and irinotecan was associated with a significant improvement in OS compared with other second-line regimens (treated with topotecan, irinotecan, paclitaxel, docetaxel, vinorelbine, or gemcitabine monotherapy), although PFS was not significantly different. These findings highlight the novelty of our study, as no large-scale prospective clinical trials have previously evaluated the combination of temozolomide and irinotecan in relapsed SCLC. By demonstrating a potential survival advantage, our results help fill an essential gap in the literature and support further investigation of this regimen as a clinically meaningful option for selected patients [13,14,15].

Our study, to our knowledge, is the first real-world cohort analysis directly comparing the combination of temozolomide and irinotecan with other second-line treatment regimens for progressed SCLC. We found that the median OS in the TMZ+IRI group was 25 months, which is significantly longer than the survival outcomes reported with both topotecan and lurbinectedin in the past [4,5]. These results suggest that TMZ+IRI could serve as a clinically meaningful alternative for patients who are appropriately selected.

Preclinical and clinical data support the mechanistic rationale for TMZ plus IRI. Temozolomide, an alkylating agent that can cross the blood–brain barrier, has demonstrated activity against brain metastases [10,11]. Irinotecan, a topoisomerase I inhibitor, has proven efficacy in both first-line and salvage settings [9,11]. Moreover, synergy between TMZ and IRI has been confirmed in preclinical studies [16]. Consistent with recent large—population studies, we observed that the presence of brain metastases was independently associated with worse OS, underlining the clinical importance of regimens with CNS activity [17]. In multivariate analysis, we found that both the presence of brain metastases and the use of alternative second-line treatments independently predicted poor OS. Our results are consistent with recent real-world data indicating that temozolomide has demonstrated measurable clinical activity in heavily pretreated patients with relapsed SCLC, even when used as a single-agent therapy [18,19]. Interestingly, although no significant difference in PFS was observed between groups, the OS advantage of TMZ+IRI may reflect delayed disease progression or improved control of CNS metastases.

Host—related factors also emerged as significant prognostic indicators. In particular, lower BMI was associated with worse OS in univariate analysis, consistent with recent findings in SCLC showing that patients with lower BMI or poor nutritional/cachectic status have inferior outcomes [15]. Similarly, an ECOG performance status of 2 or higher correlated with inferior survival, in agreement with established prognostic models and guideline recommendations [3]. These observations highlight the crucial importance of incorporating host-related variables, such as body mass index and performance status, into clinical decision-making for patients with SCLC. Beyond traditional tumor-related prognostic markers, patient-specific factors reflecting overall functional reserve, nutritional status, and comorbidity burden can substantially influence treatment tolerance, survival outcomes, and quality of life. Therefore, careful assessment of these host-related characteristics is crucial to optimize therapeutic choices, inform individualized treatment planning, and ultimately enhance both the efficacy and safety of second-line interventions in this challenging patient population.

This study has several limitations. Its retrospective design, single-center setting, and small sample size restrict the generalizability of the findings and preclude more detailed subgroup analyses. Another significant limitation is the heterogeneity of the comparator group, which included a range of second-line regimens, such as topotecan, irinotecan, paclitaxel, docetaxel, vinorelbine, and gemcitabine. This variability may have influenced treatment outcomes and affected the comparison with TMZ+IRI. Additionally, toxicity and quality-of-life data were not systematically collected, which prevented a comprehensive assessment of tolerability. Despite these limitations, the substantial overall survival benefit observed in the TMZ+IRI group provides meaningful real-world evidence supporting the potential role of this regimen in relapsed SCLC.

### Clinical Implications and Future Directions

Given the limited progress in SCLC beyond first-line therapy, TMZ-based combinations warrant further prospective evaluation. Identifying predictive biomarkers such as MGMT promoter methylation could help select patients most likely to benefit from TMZ [19,20]. Larger prospective clinical studies are needed to confirm these findings and optimize sequencing strategies.

## 5. Conclusions

In this retrospective cohort study, the combination of temozolomide and irinotecan as second-line treatment for SCLC showed a statistically significant improvement in OS compared to other treatment regimens. Patients treated with TMZ+IRI had a median OS of 25 months versus 8 months in the control group (*p* = 0.002), and one-year survival was significantly higher (58.2% vs. 25.4%). In multivariate analysis, the presence of brain metastases and the use of alternative second-line therapies were independently associated with worse survival outcomes. Although PFS was similar between groups, the TMZ+IRI regimen demonstrated a favorable trend in disease control, with a comparable 6-month PFS rate. Additionally, a higher number of total treatment lines was associated with an increased risk of disease progression. These findings suggest that temozolomide plus irinotecan may offer a survival benefit as a second-line option in selected SCLC patients, particularly those with a good performance status and manageable comorbidities. Further prospective, large-scale randomized studies are needed to validate these results and identify the optimal patient population for this treatment approach.

## Figures and Tables

**Table 1:** Demographic and Clinical Characteristics of Patients According to Second-Line Treatment Groups (temozolomide + irinotecan vs. other regimens).

Variable	Temozolomide + Irinotecan n (%)	Other Regimens, n (%)	*P*
Sex, n (%) Male	21 (87.5%)	11 (84.6%)	1.000
Female	3 (12.5%)	2 (15.4%)	
Age, mean ± SD (range)	59.8 ± 7.0 (43–73)	59.5 ± 8.4 (46–75)	0.899
BMI, mean ± SD (range)	27.9 ± 4.5 (20.3–38.3)	24.6 ± 3.5 (18–30)	**0.033**
Smoking status, n (%) Smoker	14 (58.3%)	9 (69.2%)	0.264
Non-smoker	0 (0.0%)	1 (7.7%)	
Former smoker	10 (41.7%)	3 (23.1%)	
Smoking, pack-years	42.5 (30.5–75)	50 (40–50)	0.906
ECOG 2, n (%)	3 (12.5%)	4 (30.8%)	0.213
Liver metastases, n (%)	8 (33.3%)	4 (30.8%)	1.000
Lung metastases, n (%)	14 (58.3%)	10 (76.9%)	0.305
Bone metastases, n (%)	16 (66.7%)	6 (46.2%)	0.300
Brain metastases, n (%)	11 (47.8%)	5 (38.5%)	0.731
Other metastases, n (%)	8 (33.3%)	2 (15.4%)	0.283
Radiotherapy, n (%)	4 (16.7%)	3 (23.1%)	0.678
First-line therapy, n (%) Platinum-Etoposide	23 (95.8%)	13 (100%)	1.000
Platinum-Etoposide atezolizumab	1 (4.2%)	0 (0.0%)	
Total number of treatment lines, median (IQR)	2.5 (2–3)	2 (2–3)	0.902
Follow-up duration, months, median (IQR)	13.5 (7.25–24.75)	7 (3,5–13)	0.062
Progression, n (%)	12 (50.0%)	6 (46.2%)	1.000
Death, n (%)	12 (50.0%)	12 (92.3%)	0.013

ECOG: performance status, BMI: body mass index, IQR: interquartile range, SD: standard deviation. Other regimens: topotecan, irinotecan, paclitaxel, docetaxel, vinorelbine, or gemcitabine. Bold values indicate statistically significant results (*p* < 0.05).

**Table 2:** Overall survival outcomes according to second-line treatment regimen.

		Median Overall Survival					
		Months		SE	95% CI		
					Min		Max
***p* = 0.002**	Temozolomide + Irinotecan	25		7.2	10.9		39.1
	Other regimens	8		1.7	4.7		11.3
	Overall	12		1.7	8.7		15.3
		**Overall survival rate**					
		1-year	2-years			3-years	
	Temozolomide + Irinotecan	58.2%	53.3%			35.6%	
	Other regimens	25.4%	-			-	
	Overall	47.4%	32.4%			21.6%	

Other regimens: topotecan, irinotecan, paclitaxel, docetaxel, vinorelbine, or gemcitabine, SE: standard error; CI: confidence interval.

**Table 3:** Predictors of Overall Survival: Univariate and Multivariate Cox Regression Analyses.

Variable	Univariate Analysis			Multivariate Analysis		
	HR	95% CI	*p*	HR	95% CI	*P*
Sex (Male vs. Female)	1.509	0.446–5.109	0.508			
Age at diagnosis	1.027	0.968–1.090	0.376			
Smoking (Ref: Former)			0.155			
Current	1.866	0.755–4.611	0.176			
Never	7.000	0.772–63.435	0.084			
Smoking, pack-years	0.998	0.983–1.013	0.759			
BMI	0.885	0.805–0.972	**0.011**	0.910	0.815–1.016	0.094
ECOG (2 vs. 0–1)	2.203	0.868–5.589	0.096			
Liver metastases	1.148	0.488–2.699	0.752			
Lung metastases	0.784	0.318–1.934	0.598			
Bone metastases	0.833	0.369–1.880	0.659			
Brain metastases	0.543	0.233–1.269	0.159	0.370	0.144–0.949	**0.039**
Total treatment lines	0.818	0.518–1.293	0.390			
Other regimens (vs. TMZ+IRI)	3.495	1.489–8.203	**0.004**	2.820	1.030–7.722	**0.044**

HR: hazard ratio; CI: confidence interval; ECOG: eastern cooperative oncology group; BMI: body mass index; TMZ+IRI: temozolomide + irinotecan. Other regimens: topotecan, irinotecan, paclitaxel, docetaxel, vinorelbine, or gemcitabine. Bold values indicate statistically significant results (*p* < 0.05).

**Table 4:** Progression-Free Survival outcomes according to second-line treatment.

		Median Progression-Free Survival			
		Months	SE	95% CI	
				Min	Max
***p* = 0.733**	Temozolomide + Irinotecan	8	1.5	5.1	10.9
	Other regimens	7	3.8	0.0	14.4
	Overall	8	0.9	6.2	9.8
		**Progression-free survival rate**			
		6-month	1-year		
	Temozolomide + Irinotecan	62.5%	20.8%		
	Other regimens	65.0%	24.8%		
	Overall	63.2%	23.7%		

SE: standard error. Other regimens: topotecan, irinotecan, paclitaxel, docetaxel, vinorelbine, or gemcitabine, CI: confidence interval.

**Table 5:** Predictors of progression-Free Survival: Univariate and Multivariate Cox Regression Analyses.

Variable	Univariate Analysis			Multivariate Analysis		
	HR	95% CI	*p*	HR	95% CI	*p*
Sex (Male vs. Female)	0.556	0.155–1.989	0.367			
Age at diagnosis	0.985	0.911–1.065	0.703			
Smoking (Ref: Former)			0.643			
Current	0.608	0.216–1.715	0.347			
Never	0.000	0.000–0.000	0.987			
Smoking, pack-years	1.005	0.990–1.021	0.493			
BMI	1.014	0.916–1.122	0.790			
ECOG (2 vs. 0–1)	0.756	0.170–3.376	0.713			
Liver metastases	0.374	0.103–1.356	0.135	0.487	0.117–2.036	0.325
Lung metastases	0.983	0.366–2.637	0.973			
Bone metastases	0.477	0.171–1.327	0.156	0.794	0.256–2.460	0.689
Brain metastases	0.869	0.317–2.381	0.785			
Total treatment lines	1.664	1.057–2.619	0.028	1.623	0.926–2.845	0.091
Other regimens (vs. TMZ+IRI)	1.177	0.439–3.154	0.746	2.019	0.676–6.030	0.208

HR: hazard ratio; CI: confidence interval; ECOG: eastern cooperative oncology group; BMI: body mass index; TMZ+IRI: temozolomide + irinotecan.

## Data Availability

The datasets generated and/or analyzed during the current study are available from the corresponding author upon reasonable request.
